# Disentangling of Local and Wide-Field Motion Adaptation

**DOI:** 10.3389/fncir.2021.713285

**Published:** 2021-08-31

**Authors:** Jinglin Li, Miriam Niemeier, Roland Kern, Martin Egelhaaf

**Affiliations:** Neurobiology, Bielefeld University, Bielefeld, Germany

**Keywords:** optic flow, motion adaptation, fly, LPTC, local adaptation, global adaptation, electrophysiology, computational modeling

## Abstract

Motion adaptation has been attributed in flying insects a pivotal functional role in spatial vision based on optic flow. Ongoing motion enhances in the visual pathway the representation of spatial discontinuities, which manifest themselves as velocity discontinuities in the retinal optic flow pattern during translational locomotion. There is evidence for different spatial scales of motion adaptation at the different visual processing stages. Motion adaptation is supposed to take place, on the one hand, on a retinotopic basis at the level of local motion detecting neurons and, on the other hand, at the level of wide-field neurons pooling the output of many of these local motion detectors. So far, local and wide-field adaptation could not be analyzed separately, since conventional motion stimuli jointly affect both adaptive processes. Therefore, we designed a novel stimulus paradigm based on two types of motion stimuli that had the same overall strength but differed in that one led to local motion adaptation while the other did not. We recorded intracellularly the activity of a particular wide-field motion-sensitive neuron, the horizontal system equatorial cell (HSE) in blowflies. The experimental data were interpreted based on a computational model of the visual motion pathway, which included the spatially pooling HSE-cell. By comparing the difference between the recorded and modeled HSE-cell responses induced by the two types of motion adaptation, the major characteristics of local and wide-field adaptation could be pinpointed. Wide-field adaptation could be shown to strongly depend on the activation level of the cell and, thus, on the direction of motion. In contrast, the response gain is reduced by local motion adaptation to a similar extent independent of the direction of motion. This direction-independent adaptation differs fundamentally from the well-known adaptive adjustment of response gain according to the prevailing overall stimulus level that is considered essential for an efficient signal representation by neurons with a limited operating range. Direction-independent adaptation is discussed to result from the joint activity of local motion-sensitive neurons of different preferred directions and to lead to a representation of the local motion direction that is independent of the overall direction of global motion.

## Introduction

Neuronal adaptation, i.e., the adjustment of the response properties of neurons according to their recent stimulus history, is a general feature of neurons (Kohn, 2007; Rieke and Rudd, 2009). Motion adaptation in the fly visual motion pathway has been particularly well characterized in a variety of electrophysiological studies (Maddess and Laughlin, 1985; Harris, 1999; Harris et al., 2000; Neri and Laughlin, 2005; Kurtz, 2007, 2009; Kalb et al., 2008a,b; Liang et al., 2008, 2011; Nordström and O’Carroll, 2009; Liang, 2010). For the fly visual motion pathway at least two sites of adaptation have been pinpointed: at the level of retinotopically organized local motion detectors, on the one hand, and at the more downstream level of directionally selective cells with large receptive fields, the lobula plate tangential cells (LPTCs) that pool the outputs of many local motion detecting neurons, on the other hand.

From a functional perspective, adaptive processes in sensory systems often come into play when a very large stimulus range must be mapped onto the operating range of neurons limited by the underlying biophysical mechanisms. Adaptation then leads to the neuron’s working range being adjusted to the prevailing stimulus conditions. Although this functional aspect might play a role, various other functional consequences were also discussed with regard to movement adaptation. These range from the energy required for signal representation (Brenner et al., 2000; Fairhall et al., 2001; Heitwerth et al., 2005) to an enhancement of the differential sensitivity to speed, the direction of motion and discontinuities in the motion stimuli (Maddess and Laughlin, 1985; Neri and Laughlin, 2005; Liang et al., 2008; Kurtz et al., 2009b). During translatory locomotion in cluttered natural environments such velocity discontinuities may result from changes in the depth structure of the environment. Their representation in the visual motion pathway could be shown to be enhanced as a consequence of motion adaptation (Liang et al., 2008; Ullrich et al., 2015; Li et al., 2016, 2017).

The experimental evidence for all these conclusions is based for methodological reasons mainly on electrophysiological recordings from LTPCs, i.e., at a processing stage where the local movement detectors sensing motion in a retinotopic way across the entire visual field have already been spatially pooled to a large extent (reviews: Egelhaaf, 2009; Borst et al., 2020). Whereas LPTCs are accessible to electrophysiological recording because of their relatively large size, this is hardly possible in a systematic way for their small local motion-sensitive input neurons (Gruntman et al., 2018). Therefore, most inferences about motion adaptation at the level of local motion-sensitive neurons have been drawn only indirectly. Since most stimuli employed in previous studies led to adaptation at both the level of local motion-sensitive neurons and the level of LPTCs, local and wide-field adaptation mechanisms could not easily be disentangled.

Therefore, we designed a novel stimulus paradigm, which allowed us to exclude largely the effects of local motion adaptation and to compare the consequences of adaptation with motion stimuli that avoided local motion adaptation to those evoked by stimulus conditions that are identical in all other aspects, except that local motion adaptation was not excluded. In this way, we could pinpoint by intracellular recording from a prominent LPTC, the HSE-cell (Hausen and Egelhaaf, 1989), for the first time the adaptive effects caused at the level of LPTCs, as well as, by comparing the responses obtained with and without local adaptation, the adaptive effects that are a consequence of local motion adaptation. The experimental results will be interpreted on the basis of a computational model of the fly’s visual motion pathway that has been proposed in previous studies (Li et al., 2016, 2017), but is now extended to include motion adaptation at both the level of local movement detectors as well as the LPTCs.

## Materials and Methods

### Visual Stimulation

**FIGURE 1:**
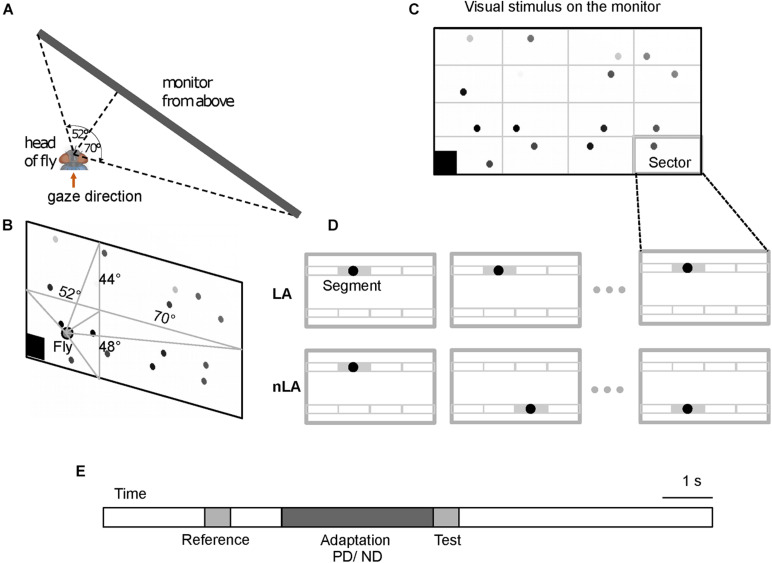
Schematic of visual stimulation paradigm. **(A,B)** Schematic of the monitor screen as seen from the perspective of the fly from above **(A)** and from behind **(B)**. The monitor is placed in front of the fly’s right eye. The perpendicular from the monitor hitting the fly’s right eye at its smallest distance to the monitor divides the monitor screen into four parts. The angles indicate the angle at which the left and right as well as the upper and lower edges of the screen are seen from the fly’s perspective. 16 horizontally moving dots constitute the visual stimulus. **(C)** One frame of a visual stimulus as an example. The stimulus consisted of a white background and 16 dots with different gray levels from white to black. The black square at the bottom left corner of the screen was a trigger signal for data acquisition. The gray grid that divides the screen into 16 identical sectors was invisible to the fly and is shown here just to illustrate the sector of the screen in which each dot was allowed to move. **(D)** Schematic of stimulus conditions with local motion adaptation (LA) and without local motion adaptation (nLA) demonstrated for one sector shown at consecutive instants of time as an example. Thin gray lines within the sector outline eight segments that a dot possibly traverses during the stimulation period. The gray segment is the active segment the dot is traversing at the corresponding time indicated by the time axis. Under the LA condition the dot repeatedly moves across the same segment again and again for the entire stimulation period to evoke local motion adaptation, while under the nLA condition the dot is repeatedly relocated to move in a different segment and, thus, to avoid eliciting local motion adaptation. **(E)** Adaptation protocol showing the time sequence of the reference, adaptation, and test stimuli. During adaptation, the dots moved in either the preferred direction (PD, left to right on the screen) or the anti-preferred direction (ND, right to left on the screen) of the right HSE-cell (see Materials and Methods for details).

Stimulus movies were presented to the animal on a monitor (Acer LCD monitor GN246HL) at a rate of 144 fps and a luminance of white and black stimulus areas of 250 and 2 cd/m^2^, respectively. To stimulate the right eye, the monitor was placed at the right side of the animal. The perpendicular from the fly’s eye to the monitor screen was 12.7 cm and virtually divided the screen into four quadrants of different size (for the angular size and location of the monitor screen from the perspective of the fly see Figures 1A,B). The stimulus field was located well within the large receptive field of the HSE-cell (Hausen, 1982b; Krapp et al., 2001). The position and the orientation of the fly were adjusted according to the fly’s deep pseudopupil (Franceschini, 1975). The stimulus movies were generated with a custom program written in Matlab, 2017b (The Mathworks, Inc., Natick, MA, United States) and displayed by the command line version of the VLC media player^1^ under control of custom scripts on Ubuntu Linux.

To investigate the impact of adaptive motion on the response gain of the HSE-cell, a 500 ms reference and a 500 ms test stimulus were presented before and after a 3 s adaptation motion stimulus, respectively. A 1 s interval without any motion separated the end of the reference stimulus and the start of the adaptation period; the last frame of the reference stimulus was constantly shown in this interval. In contrast, the test stimulus directly followed the adaptation period (Figure 1E). Two seconds before and 5 s after the reference and test stimulus, respectively, neuronal responses were recorded with the stimulus pattern stationary so that the adaptive effects in the cell can subside.

To disentangle the adaptive effects caused by local motion adaptation from those caused by global adaptation, we designed two types of motion stimuli, which had identical overall stimulus strength, but only differed in the adaptation they induced: one elicited local motion adaptation (LA conditions), while the other avoided local motion adaptation (nLA conditions). This means that it had to be ensured that in the LA case the same areas in the visual field were repeatedly stimulated and in the nLA case exactly that had to be prevented, whereby the overall stimulus was not allowed to differ—apart from statistical details. This was accomplished by subdividing for both stimulus types the 3 s adaptation period into six consecutive 500 ms time intervals and by using stimulus patterns composed of black dots on a white background (Figures 1B–D), with all dots moving in small segments in the same direction and with the same speed. Both types of stimuli comprised the same number of dots and the same dot velocity, i.e., both types of stimuli were characterized at each instant of time by the same overall strength of motion. The only difference was that under the LA condition, dots moved repeatedly across the same small segments (Figure 1D upper diagrams), while under the nLA condition dots moved always across different segments in successive 500 ms intervals without trajectory overlap during the entire stimulus period (Figure 1D bottom diagrams). More precisely, the white background of the screen (1,920 px × 960 px) was invisibly subdivided into 16 equally sized sectors (480 px azimuth × 240 px elevation) (Figure 1C); each sector confined the moving area of a single dot. The sectors were subdivided into 4 by 2 invisible non-overlapping 120 px × 120 px segments (Figure 1D). A 48 px-diameter black dot moved horizontally for 500 ms within a given segment at a velocity of 240 px/s. Dot size (speed) was 7° × 7° (35 deg/s) perpendicular to the fly (0°/0°) and—as a consequence of systematic perspective distortions due to the flat monitor screen—1.6° × 2.5° (8 deg/s) at the right margin of the screen stimulating the lateral visual field (70°/0°). A dot moving repeatedly in just one of the eight randomly chosen segments allowed local adaptation to PD or ND (LA stimulus). Alternatively, a dot could move for 500 ms consecutively in one of the eight different segments of a given sector in random order and, thus, did not elicit local motion adaptation (nLA stimulus). Note that the strength of the responses induced by the individual dots in the different sectors may differ for two reasons: (a) the sensitivity gradient within the HSE-cell’s receptive field (Hausen, 1982b; Krapp et al., 2001) and (b) the decrement of retinal dot size and velocity toward the lateral part of the stimulus screen as a consequence of perspective distortions (see above). Within each sector, however, these gradients are rather small, so that there should be only negligible differences between the response strengths to dot movement in the different segments of a given sector and thus to LA and nLA adaptation.

To reduce flicker responses of the recorded neuron due to a synchronous appearance and disappearance of the different dots when they begin and end their movements, each started and ended moving at a different phase randomly chosen from 16 phases equally distributed within the 500 ms interval. The flickering effects were further reduced by gradually increasing and decreasing the gray values (black and gray dots in Figures 1B,C) of the dots in three steps during the initial and the final 90 ms of each 500 ms movement period.

For both the LA and nLA conditions, motion adaptation was either directed in the preferred direction (PD, left to right on the screen) or the null direction (ND, right to left) of the right HSE-cell. Accordingly, the fly was confronted with four different stimulus conditions: LA and nLA with motion during the adaptation period in the preferred direction and null direction, respectively. To adapt the same location for the two adaptation directions, under the PD and ND condition a dot traversed the same segment in the opposite direction. For the LA condition, the segments traversed by the dots were identical for the reference, adaptation, and test phase. Since for the nLA condition repetition of movement in the same segment was avoided, the segments that the dots traversed never matched over the entire reference, adaptation, and test phases. As a consequence, the traversed segments had to differ between the reference and test stimuli during nLA motion. Therefore, pairs of nLA motion stimuli were generated where the reference and test patterns were exchanged between consecutive stimulus presentations to ensure that the test and the reference stimulus were overall the same apart from their temporal order. Eight pairs of stimuli of different dot constellations were generated to reduce the effect of pattern-specific modulations in the average neuronal response that can be observed in LPTCs if stimuli contain low spatial frequency components (Meyer et al., 2011).

### Animal Preparation and Electrophysiological Experiments

One-day-old female blowflies (*Calliphora vicina*) from our laboratory stock were dissected according to standard procedures for intracellular recording of HSE-cells (Dürr and Egelhaaf, 1999). Briefly, a blowfly was anesthetized with CO_2_ and fixed with wax on a microscope glass slide at the dorsal thorax, the wing bases, and the abdomen. The legs were removed, and wounds were waxed. The head was pitched down and fixed to the thorax to make its rear accessible. The head capsule of both hemispheres was opened from the back, the right opening for the intracellular recording of the HSE-cell, and the left opening for the indifferent electrode. Ringer’s solution (composition in mM: NaCl 128.3, NaHCO_3_ 1.67, CaCl_2_ 1.89, KCl 4.69, Glucose 12.6, KH_2_PO_4_ 3.38, Na_2_HPO_4_ 3.3; all chemicals from Merck and Fisher Scientific, Germany) was applied *via* the indifferent electrode, which was connected *via* an electrode holder and a silicone tube to a syringe, to prevent the brain from drying out. The proboscis and the antennae were removed. Tissues bridging both hemispheres were partially removed to make the esophagus accessible, which was pulled out of the head and fixed to the dorsal part of the thorax with wax. The wounds were waxed. Air sacks, fat bodies, and part of tracheas were removed to make the axon of the HSE-cell accessible. The electrode for intracellular recording was pulled from a borosilicate thin-walled glass pipette (OD = 1.00 mm, Warner Instruments) with a Flaming/Brown Micropipette Puller (Model P-1000, Sutter Instrument). The electrodes were filled with 1 M KCl and had resistances ranging from 27 to 45 MΩ. Intracellular recordings were obtained from the axon of the right HSE-cell. The cell could be identified unambiguously by its characteristic response properties (Hausen, 1982a) using both a search stimulus on the monitor as well as a hand-held light probe. Graded changes in their membrane potential are the main response mode of HS-cells, even if recorded in their axons (Hausen, 1982a). The membrane potential was recorded with a bridge amplifier (BA-03x, npi, Germany). The membrane potential was sampled at a frequency of 25 kHz and an amplitude resolution of about 0.3 mV (National Instruments PCIe-6251, 16-bit-ADC resolution). Digitized data were collected by a program based on the Matlab data acquisition toolbox (program written by Jens-Peter Lindemann) and stored on a hard disk for offline data analysis. All experiments were carried out at temperatures ranging between 22.3 and 24.5°C.

### Data Analysis

The analysis of recorded neuronal responses, the statistics as well as the model simulations were done with programs written in Matlab, 2019b. 13 HSE-neurons from different flies were recorded (550 sweeps in total; at least between 4 and 24 trials for each of the four stimulus conditions, median number of sweeps per condition: 9). Another set of recordings using the same design, but not exactly the same stimuli, were obtained in the Ph.D. project of Li, 2019; this data led to the same overall conclusion as the more recent data obtained in the present study. The resting potential as determined by the time-averaged membrane potential over 2 s before the start of the motion stimuli was subtracted from the responses. Only cells/trials were included into the analysis, if the resting potential was more negative than −40 mV, less membrane potential drift than 1 mV between the beginning and end of the responses and if responses of at least four repetitions under the same stimulus condition could be recorded. The responses were for- and backward low-pass filtered with a time constant 180 ms to focus our analysis on the graded component of the HSE-cell response. The reference response (R_ref_) and the test response (R_test_) were calculated as time-average over the respective last 400 ms of the stimulation period, thus not taking into account the initial 100 ms response transients. Similarly, the responses at the beginning and the end of the adaptation period (R_badp_, R_eadp_) were calculated as an average over 400 ms with the averaging starting only 100 ms after motion onset. Changes in the HSE-cell response evoked by motion adaption were assessed in two ways:

(1) The relative amplitude reduction of the response during adaptation (RAR_adpt_) was calculated as the difference between average response at the end of adaptation (R_eadp_) and the average response at the beginning of the adaptation period (R_badp_) and normalized to the average reference response:

(1) $$RAR_{adpt}=\left(R_{eadpt}-R_{badpt}\right)/R_{ref}$$

(2) How much the test response was smaller than the reference response was determined in a similar way as the normalized difference between the test and reference response:

(2) $$RAR_{test}=\left(R_{test}-R_{ref}\right)/R_{ref}$$

To determine the time constant for the decay in the response amplitude during the adaptation period, the HSE-cell responses were fitted by the formula

(3) $$R_{HSE}=a^{*}exp\left(-t/\tau \right)+b$$

with *R*_*HSE*_ being the average membrane potential of 13 HSE-cells, *t* the time, τ the approximated time constant, and *a*, *b* are parameters for curve fitting with the scaling factor *a* and *b* the level where the exponential curve settles.

### Modeling

The experimental data were interpreted on the basis of a computational model of the visual motion pathway of flies, which has been developed in two previous studies (Li et al., 2016, 2017) apart from one extension accounting for the adaptation properties of the HSE-cell. This kind of computational model, which has a long tradition in the field of insect motion vision (Borst and Egelhaaf, 1989; Borst, 2000), is not intended to mimic the cellular circuits that could be identified in recent years through sophisticated anatomical, molecular, and genetic approaches often combined with functional imaging (Mauss et al., 2017; Yang and Clandinin, 2018; Borst et al., 2020). Rather, the aim was to functionally characterize the crucial computational processes at each of the stages of the visual motion pathway. A one-to-one mapping of these processing steps to specific cellular elements is neither intended nor appropriate.

**FIGURE 2:**
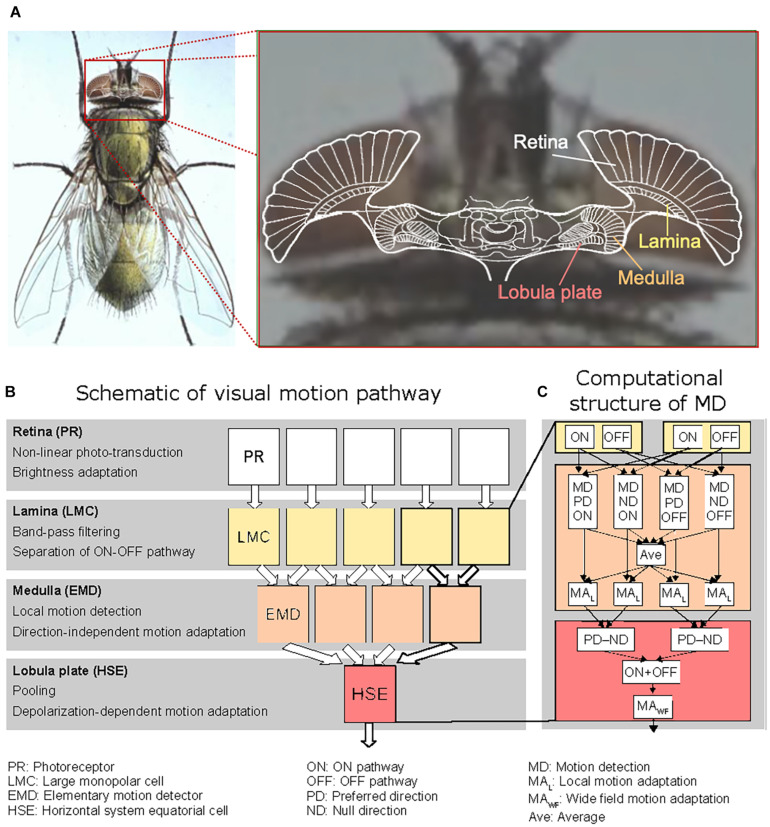
Organization of the processing stages of the fly’s visual motion pathway. **(A)** Sketch of a horizontal section of the brain of the blowfly projected onto a photograph of its head, with the retina and the three main visual neuropiles labeled. The colors correspond to those used to mark the corresponding processing stages in the computational model shown in panels **(B,C)**. **(B)** Schematic illustration of the visual motion pathway with its consecutive processing stages and its retinotopic columnar organization (up to the level of the Lobula plate). The computations performed at the individual processing stages are briefly characterized at the left of the diagram. **(C)** Sketch of the different computations performed in two neighboring channels of the model Lamina and of the motion detection (MD) processing in the Medulla, as well as the computations performed at the HSE level. The non-linearly transformed photoreceptor signals are temporally band-pass filtered in each Lamina column by the Large Monopolar Cells (LMC), half-wave rectified and split into an ON and an OFF channel. The Lamina output signals are fed into a model of an elaborated elementary movement detector (EMD) of the correlation type. The signals are processed separately at the EMD stage for the ON and OFF channels as well as for preferred direction (PD) and null direction motion (ND), respectively. For motion adaptation, the outputs of the resulting four channels, shown here for horizontal motion, are averaged (AVE); the average also includes the signals of the corresponding four vertically oriented movement detecting elements (not shown) leading to a direction-independent signal representing the overall motion at the respective location in the visual field. Local motion adaptation (MA_L_) is accomplished by dividing the outputs of the movement detection channels by the averaged motion signal after low-pass filtering. The movement detector channel outputs are then summated according to their respective sign (PD positive, ND negative) by the model HSE-cell. For the details of the different computational steps, see Materials and Methods.

According to the overall structure of the fly visual system, the model of the visual motion pathway is composed of successive layers of retinotopic arrays of model photoreceptors (PRs), the lamina with its characteristic large monopolar cells (LMCs), the medulla with the elementary motion detectors (EMDs), and the lobula plate with a characteristic LPTC, the HSE-cell, integrating the output signals of a large array of EMDs (Figure 2). The model parameters were tuned in the previous studies by a systematic search to qualitatively capture adaptive features revealed in previous electrophysiological studies (Laughlin and Hardie, 1978; Maddess and Laughlin, 1985; Juusola et al., 1995; Harris et al., 2000; Kurtz et al., 2009b) as well as the experimental results presented in the present study.

The peripheral visual system of the model consisted of PRs with low-pass-like temporal characteristics and the LMCs with their high-pass-like temporal characteristics. It was modeled according to Li et al. (2016). The LMC output was half-wave rectified and split into an ON and an OFF pathway according to its biological counterparts in the fly visual system (Figure 2C; Haag et al., 2017). Brightness adaptation of the photoreceptors was accomplished essentially by dividing a fast signal branch representing fast fluctuations in the input signal by a slow signal branch representing signal fluctuations on a much slower timescale. The fast and slow time constants amounted to 9 and 250 ms, respectively.

In the medulla, the outputs of the ON and OFF pathways, respectively, were fed into the adaptive motion detector model. This motion detector model was of the correlation-type consisting of two mirror symmetric half-detectors sensitive to motion in opposite directions (Figure 2C). Motion adaptation was implemented at the level of the half-detector output by a mechanism similar to that of brightness adaptation of the photoreceptors (see above), i.e., by dividing a fast signal branch representing fast fluctuations in the motion signal by a slow signal branch representing pattern velocities on a much slower timescale. Since motion adaptation takes much longer than brightness adaptation in the peripheral visual system, the “fast” and “slow” time constants were chosen to be much larger, 20 and 4,000 ms, respectively, than the time constants characteristic of brightness adaptation (see above). While the fast branches were the different half-detector outputs after being low-pass filtered with a small time constant, the slow branch was the average of the outputs of the PD and ND half-detectors of both the ON and OFF pathways after being low-pass filtered with a large time constant, leading to a direction-independent motion adaptation (Figure 2C). Except for two parameters, all parameter settings characterizing the peripheral visual system (photoreceptors and LMCs) as well as the adaptive local motion detector are identical to those as specified in the legend of Figure 1 in Li et al. (2017). To obtain a slightly better fit with the electrophysiological data recorded in the current study, just two parameters determining the speed of recovery and the strength of local adaptation were further adjusted: p1 = 50 ms (instead of 30 ms) and p2 = 500 ms (instead of 150 ms) (Li et al., 2017).

In the LPTC model, for simplicity, we assumed that the outputs of the local motion detectors are linearly summated in the lobula plate. Here, both half-detectors, i.e., ON and OFF, responding best to preferred-direction motion of the LPTC contributed to the sum with a positive sign, whereas both half-detectors responding best to null-direction motion of the LPTC contributed with a negative sign (Figure 2C). The simplification of linearly summating the motion detector outputs instead of implementing a dynamic gain control at this processing stage (Borst et al., 1995; Lindemann et al., 2005) is justified in the context of the current paper, since the pattern size in all model simulations was kept constant. Adaptation at the LPTC level was concluded in a previous study (Kurtz, 2007) to be directionally selective and elicited exclusively during depolarization of the cell and mimicked here in a computationally parsimonious way by subtracting from the summated EMD responses (R_EMD_) a low-pass filtered version of them. During hyperpolarization the summated EMD response was just scaled to the membrane potential as has been measured in the experiment:

(4) RHSE={FmV*Fratio*(∑REMD-Fadapt*LPF(max(∑REMD,0)))for∑REMD≥0FmV*∑REMDfor∑REMD<0}

with F_mV_ representing the scaling factor transforming the model response into mV, and *F*_*ratio*_ representing the ratio between the asymmetric depolarizing and hyperpolarizing response amplitude—as averaged across all neurons used for our analysis—to a given stimulus moving in the preferred and null direction, respectively. *F*_*mV*_ = 842 and *F*_*ratio*_ = 1.97 were determined by scaling the *R*_*ref*_ of the model under nLA conditions to that of the cell. The time constant of the first-order low-pass filter (indicated by LPF in eq.4) was 1,170 ms as determined from the neural responses averaged across all cells used for our analysis under the condition without local motion adaptation (see Eq. 3). *F*_*adapt*_ = 0.32, the scaling factor for the magnitude of the adaptive decay was determined by scanning through the parameter space in steps of 0.01 for the minimal difference between the model response and the cell response.

## Results

To pinpoint the role of local vs. wide-field motion adaptation for shaping the responses of LPTCs we specifically designed a visual stimulus paradigm, which allowed us, while stimuli being identical with respect to their overall motion strength, to include (LA) or exclude (nLA) local adaptation. The graded membrane potential changes of the HSE-cell, recorded intracellularly under our nLA conditions were, therefore, shaped only by wide-field motion adaptation at the level of this LPTC. However, since wide-field motion adaptation cannot be avoided when analyzing motion adaptation on the basis of HSE-cell responses, the responses obtained under the LA conditions were not only the consequence of local motion adaptation, but inevitably also of wide-field adaptation. Therefore, we inferred the characteristics of local motion adaptation presynaptic to the HSE-cell indirectly by comparing the corresponding responses obtained under nLA and LA conditions.

**FIGURE 3:**
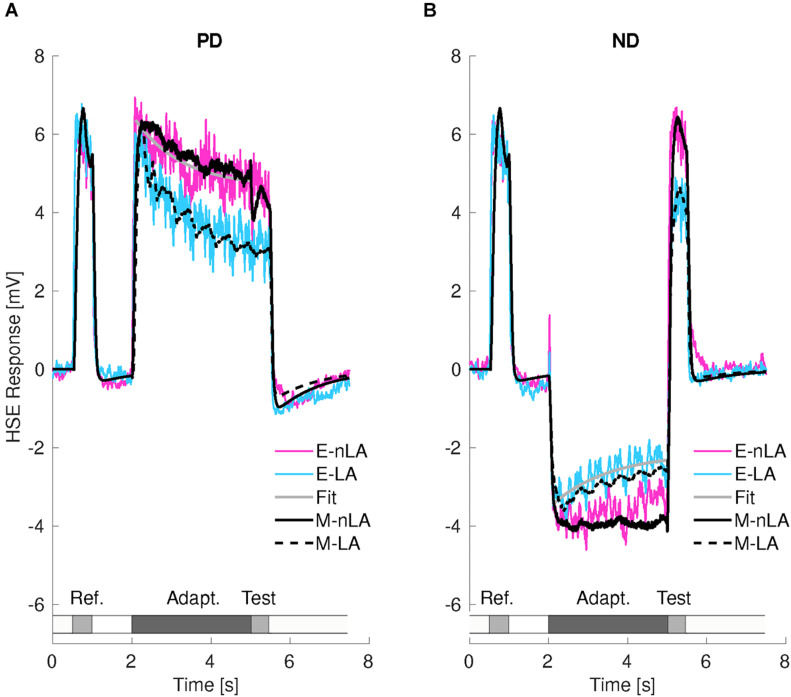
Consequences of motion adaptation for the HSE-cell and the adaptive model of the visual motion pathway. **(A)** Average time-dependent responses of 13 HSE-cells for preferred direction (PD) motion with local motion adaptation (LA, cyan trace) and without local motion adaptation (nLA, magenta trace). Smooth gray line is the fitted curve used for estimating the time constant of wide-field motion adaptation (see Methods). The black traces represent the corresponding responses of the adaptive model of the visual motion pathway. The stimulation protocol is schematically illustrated below the cell response. **(B)** Same as in panel **(A)**, however, for null direction (ND) adaptation. Smooth gray line is the fitted curve used for estimating the time constant of local motion adaptation (see section “Materials and Methods”). The resting potential was subtracted from the responses for the experimental results, which were for- and backward low-pass filtered with a time constant of 180 ms.

Figure 3 displays the average time-dependent membrane potential changes of 13 HSE-cells for LA or nLA motion either in the preferred (PD, Figure 3A) or null (ND, Figure 3B) direction. The experimentally determined response curves are superimposed by the corresponding model responses. Since under both stimulus conditions, LA and nLA, the reference stimuli were apart from statistical differences the same, the HSE-cell and its model analog depolarized in a very similar way during the reference phases.

**FIGURE 4:**
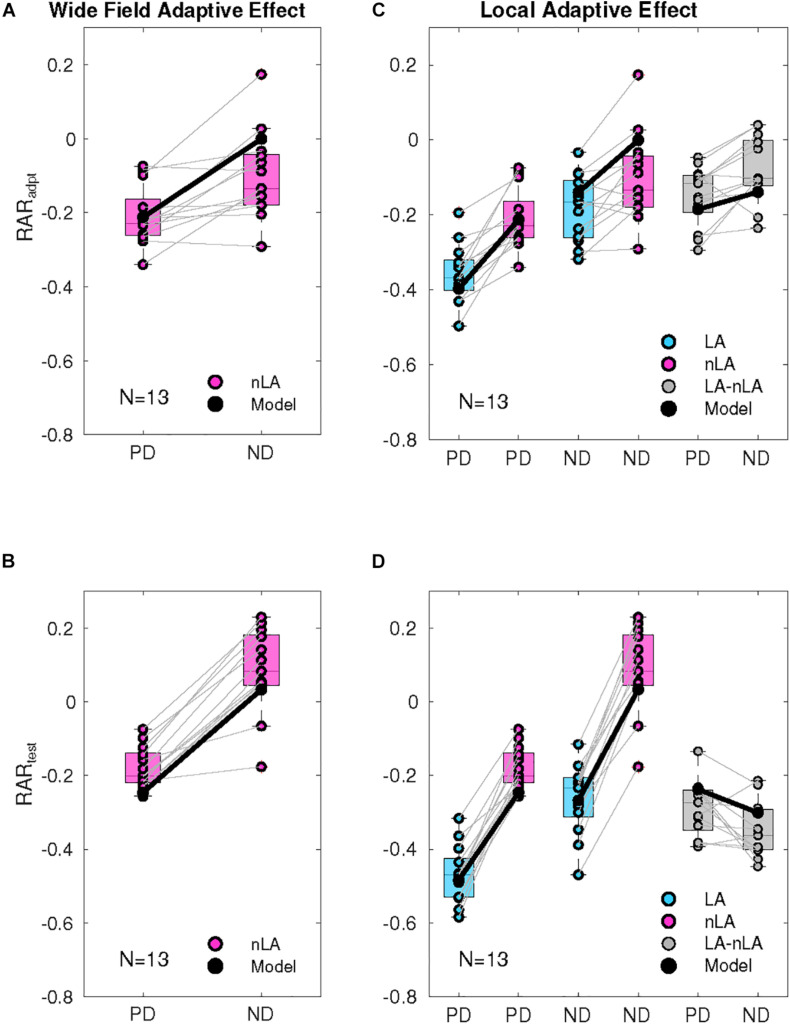
Quantification of wide-field and local adaptation. **(A)** Relative amplitude reduction of the response during motion adaptation (RAR_adpt_): Consequences of wide-field adaptation determined by comparing the response at the end and beginning of the adaptation phase. The response level at the beginning and end of the wide-field motion adaptation period (nLA) was determined by averaging over 400 ms periods starting 100 ms after the start of the adaptation period or terminating at its end. The response decrement during the adaptation period was normalized by the average reference response. The model responses (black dots) were treated in the same way. The adaptive effects are shown both pairwise for PD and ND adaptation of all 13 cells (magenta dots) and as box plots (mid-line, median, magenta box, 25–75 percentile). **(B)** Consequences of wide-field adaptation determined by comparing the test and reference responses and normalizing them to the average reference response. The averages were taken for the last 400 ms of the reference and test response, respectively. The first 100 ms were not included because of the initial response transient of the reference response. The model responses (black dots) were treated in the same way. The adaptive effects are shown both pairwise for PD and ND adaptation (nLA) for all 13 cells (magenta dots) and as box plots (mid-line, median, magenta box, 25–75 percentile). **(C)** Consequences of local adaptation during the adaptation period by comparing the responses obtained for both nLA (magenta) and LA (cyan). The responses under the LA condition were determined in the way as described in panel **(A)** for the nLA condition. The model responses (black dots) were treated in the same way as the experimentally determined responses. The adaptive effects for the LA and nLA conditions are shown pairwise [nLA: same data as in panel **(A)**] and as box plots (mid-line, median, box, 25–75 percentile). The data for PD adaptation are shown in the left, those for ND adaptation in the middle. On the right, the corresponding differences between the adaptive effects obtained under the LA and nLA conditions, respectively, are shown in gray along with the model equivalents for both PD and ND adaptation. **(D)** Consequences of local adaptation by comparing the corresponding test and reference responses obtained for both nLA (magenta) and LA (cyan). The adaptive effects were normalized to the average reference response. The responses under the LA condition were determined in the way as described in panel **(B)** for the nLA condition. The model responses (black dots) were treated in the same way as the experimentally determined responses. The adaptive effects for the LA and nLA conditions are shown pairwise [nLA: same data as in panel **(B)**] and as box plots (mid-line, median, box, 25–75 percentile). The data for PD adaptation are shown in the left, those for ND adaptation in the middle. On the right, the corresponding differences between the adaptive effects obtained under the LA and nLA conditions, respectively, are shown in gray along with the model equivalents for both PD and ND adaptation.

During PD motion adaptation (Figure 3A) the responses declined on median by slightly more than 20% even if local adaptation was prevented (nLA, magenta curve; Figure 4A, left). The decay in the response amplitude during PD adaptation under the nLA condition can be interpreted as reflecting the *time constant of wide-field adaptation*; it was estimated on this basis and approximates 1.17 s (see gray curve in Figure 3A). The consequences of PD adaptation were also reflected by an on median about 20% smaller test response in comparison to the reference response (Figure 3A, magenta curve; Figure 4B, left). The median response decay during LA is about 36% (Figure 3A, cyan curve) and thus significantly larger than during nLA (Figure 4C, left; Wilcoxon Rank Sum Test, *p* = 3.31 × 10^–4^ < 0.001). Accordingly, also the test response for LA (Figure 3A, cyan curve) is much smaller for the LA compared to the nLA condition (Figure 4D, left; Wilcoxon Rank Sum Test, *p* = 1.65 × 10^–5^ < 0.001). This difference between the HSE-cell responses obtained under the nLA and LA conditions, respectively, reflects the consequences of local motion adaptation. The time-dependent response profiles and mean adaptation-dependent response changes of the HSE-cell are qualitatively well reflected by the corresponding model responses (Figures 3A, 4, black curves/symbols). Note, that the periodic modulations in the model responses to LA are due to sensitivity gradients within the areas stimulated by the moving dots. Since during nLA they are presented in each trial in different parts of the overall stimulation area, these modulations are largely averaged out (for details on pattern-dependent response modulations in LPTCs, see Meyer et al., 2011). In the experimental data, these relatively small pattern-dependent response modulations are largely camouflaged by noise.

During adapting motion in the ND (Figure 3B), the HSE-cells hyperpolarized compared to their resting potential. With local motion adaptation being largely prevented (nLA) the hyperpolarization declined during the 3 s time interval on median by about 14% (Figure 3B, magenta curve; Figures 4A,C center). If the HSE-cell was adapted with ND motion of the same overall strength, but now allowing for local motion adaptation, the hyperpolarizing response amplitude decreased on median by about 17% (Figure 3B, cyan curve; Figure 4C center) and thus more than without local adaptation (Wilcoxon Rank Sum Test, *p* = 0.014 < 0.05). Without local motion adaptation it even increased slightly by about 7% in relation to the reference phase (Figure 3B, magenta curve; Figures 4A,B) and decreased on median by about 23% as a consequence of LA (Figure 3B, cyan curve; Figure 4D center). Consequently, the reference and test response after ND motion adaptation with and without local motion adaptation differed much (Wilcoxon Rank Sum Test, *p* = 2.08 × 10^–5^ < 0.001).

Since the effects of PD adaptation are much larger than those of ND adaptation for the nLA condition (Figures 4A,B; Wilcoxon Rank Sum Test: response decline during motion adaptation *p* = 7.13 × 10^–4^ < 0.001, test response *p* = 9.72 × 10^–5^ < 0.001), it is suggested that wide-field motion adaptation is strongly direction dependent. This might be a direct or indirect consequence of the depolarization level of the cell. In contrast to wide-field motion adaptation, which can be characterized with our stimulus paradigm largely uncontaminated by local motion adaption, this is not possible for local motion adaptation. As a proxy for the properties of local adaptation, we determined the difference in adaptive effects between LA and nLA stimulation based on the simplifying assumption that the two adaptive components superpose linearly (Figure 4, gray boxes). Since the differences are relatively small for PD and ND adaptation, although they differ significantly for the test responses (Wilcoxon Rank Sum Test: response decline during motion adaptation *p* = 0.41 i.e., nonsignificant; test response *p* = 0.008 < 0.01), local motion adaptation can be suggested to be largely independent of the direction of motion, unlike the highly direction dependent wide-field motion adaptation. since there was only little wide-field ND motion adaptation, if local adaptation was largely prevented (Figure 3B, magenta curve), the decay in the response amplitude during ND adaptation under the LA condition (Figure 3B, cyan curve) was essentially due to local motion adaptation. Therefore, the *time constant of local motion adaptation* can be estimated under this stimulus condition (see Methods). The time constant was estimated as 1.47 s (gray line in Figure 3B).

For both PD and ND motion adaptation as well as the corresponding nLA and LA conditions, the model responses (black lines in Figure 3 and black dots in Figure 4) match their experimental counterpart at least qualitatively. This finding indicates that the relatively simple computational model of the fly visual motion pathway with its two adaptive stages is sufficient to account for most of our experimental data. The only difference is that ND nLA leads to a slight decrease in response in the HSE-cell, i.e., it gets less hyperpolarized, which is not reflected in the model because of the assumption of strict directionality of wide-field adaptation (compare black and magenta lines in Figure 3B).

## Discussion

Motion adaptation in insects has been proposed to be a multi-stage process taking place at several levels in the visual motion pathway. The two most prominent adaptation stages are the array of retinotopically organized local motion-sensitive elements and their postsynaptic wide-field lobula plate tangential cells (LPTCs) that pool the outputs of local motion-sensitive cells from large parts of the visual field (Hausen, 1984; Egelhaaf, 2009, 2020; Borst, 2014). By intracellularly recording the graded membrane potential changes of a particular LPTC, the HSE-cell, we could unravel the role of the individual consecutive adaptation stages and their contribution to overall motion adaptation as it manifests itself at the level of LPTCs. This could only be achieved by using a novel stimulus design that allowed the local movement adaptation to be either included or excluded while the total stimulus strength was kept constant. Thus, wide-field adaptation could be analyzed either in isolation or together with local adaptation, the characteristics of which could then be indirectly inferred by comparing the cellular responses to the two stimulus conditions. Thus, our study is distinguished from previous studies of motion adaptation based on LPTC recordings (Maddess and Laughlin, 1985; de Ruyter van Steveninck et al., 1986; Borst and Egelhaaf, 1987; Harris et al., 2000; Reisenman et al., 2003; Neri and Laughlin, 2005; Kalb et al., 2008a) that could only characterize the joint effects of the different consecutive adaptation processes. Our results suggest, in line with previous studies (Harris et al., 1999; Kurtz et al., 2000, 2009a; Kurtz, 2007), that wide-field motion adaptation strongly depends on the motion direction. We further concluded that, in contrast, local motion adaptation is largely direction independent. We hypothesize that this independence aims at a robust representation of the direction of local motion, independent of local motion history, by the population activity of local motion-sensitive neurons with different preferred directions at a given point in the retinotopic array.

### Relation to Previous Research

Various aspects of motion adaptation have been characterized in other contexts and with different types of stimuli leading to a variety of interpretations. How do these concepts relate to our conclusions? Harris et al. (2000) suggested that motion adaptation induces three components of contrast gain modulation: (1) a contrast gain reduction as described by a shift of the contrast-response function toward higher contrasts, (2) a subtractive effect leading to a hyperpolarizing shift of the contrast-response function, and (3) a contraction of response range. Further, Harris et al. (2000) provided evidence that the contrast gain reduction was independent of the motion direction of the adapting stimulus. Based on our results, this phenomenon can be concluded to be caused by local motion adaptation. In principle, local adaptation can take place at different stages of the visual motion pathway. Since, according to Harris et al. (2000), flicker stimulation induced a much weaker contrast gain reduction than motion in any direction, contrast gain reduction is probably located after the computation of motion. Contrast gain reduction may, in agreement with our model simulations (see also Li et al., 2017), involve the pooled outputs of different subtypes of the local motion-sensitive neurons with different preferred directions to adapt each subtype equally. The adaptive reduction of the response amplitude to constant-velocity motion goes along with an enhancement of the sensitivity to velocity transients (Kurtz et al., 2009a), which has been suggested to be the consequence local adaptive mechanisms (Maddess and Laughlin, 1985) and is probably based on the process described by Harris et al. (2000) as contrast gain control. We accounted for this adaptive feature by direction independent local motion adaptation. Both the contrast gain reduction and the enhancement of the sensitivity to velocity transients could be modeled by a specific local motion adaptation mechanism (Li et al., 2017). Whether further adaptive effects of enhancing the sensitivity to other stimulus transients resulting from sudden contrast, orientation, or spatial frequency changes (Kurtz et al., 2009b) can also be explained by this mechanism needs to be further investigated. The subtractive, hyperpolarizing shift induced by motion adaptation is highly direction-dependent (Harris et al., 1999, 2000) corresponding well to the wide-field motion adaptation at the level of the HSE-cell we revealed in our study. This hyperpolarizing shift is likely associated with Ca^2+^ accumulation in the dendrite of the HSE-cell (Kurtz et al., 2000; Kurtz, 2007) and caused by the opening of specific potassium channels (Kurtz et al., 2009a).

In the previous studies, contrast gain modulation induced by motion adaptation has been assessed by varying the pattern contrast of the reference and the test stimuli presented before and after a strong preferred direction adaptation and by comparing the response amplitude for each contrast condition before and after adaptation (Harris et al., 2000). The directional gain modulation has been determined in a similar way by altering the motion direction of the reference and the test stimuli and comparing the response amplitudes before and after motion adaptation (Neri and Laughlin, 2005; Kalb et al., 2008a). Whether the adaptive components that contribute to the contrast gain modulation (Harris et al., 2000) can explain without further assumptions the local and global adaptive effects in directional sensitivity (Neri and Laughlin, 2005) needs to be clarified. Our novel stimulus paradigm allows us to further investigate the parameter space of pattern contrast and motion direction to explicitly address these issues in order to support further understanding of the mechanisms and potential functional significance of motion adaptation.

While motion adaptation has originally been suggested to be a consequence of the adaptation of the motion detector time constant (de Ruyter van Steveninck et al., 1986; Clifford and Langley, 1996), later experimental studies (Harris et al., 1999) did not support this suggestion. In a previous study (Li et al., 2017) we suggested a motion adaptation model based on a similar adaptation mechanism as accounting for brightness adaptation in the peripheral visual system (Li et al., 2016). This mechanism can reproduce direction-independent reduction of response sensitivity of local motion-sensitive neurons without causing a change in the motion detector time constant (Li et al., 2017).

### Functional Significance of Motion Adaptation

Motion adaptation in the insect visual motion pathway has been studied mostly based on recordings of wide-field motion-sensitive LPTCs. These studies used either relatively simple system-analytical stimuli (Maddess and Laughlin, 1985; de Ruyter van Steveninck et al., 1986; Borst and Egelhaaf, 1987; Harris et al., 1999, 2000; Neri and Laughlin, 2005; Kalb et al., 2008a; Kurtz et al., 2009b; Nordström and O’Carroll, 2009), or more naturalistic motion stimuli (Liang et al., 2008, 2011). Motion adaptation has usually been considered to be beneficial for enhancing the sensitivity to changes in the speed (Maddess and Laughlin, 1985), direction (Neri and Laughlin, 2005), or other stimulus or environmental features (Liang et al., 2008; Kurtz et al., 2009a). As a consequence of this kind of adaptation (Neri and Laughlin, 2005), information about the absolute value of the relevant stimulus feature (e.g., velocity) is lost for the benefit of an increased sensitivity to stimulus changes. Further studies demonstrated based on information-theoretical methods how motion adaptation facilitates efficient information transmission (Brenner et al., 2000), saves coding energy without losing information (Heitwerth et al., 2005), or even adapts without the cost of losing information about the absolute value of the prevailing level of the corresponding parameter (Fairhall et al., 2001). These functional benefits of motion adaptation are very similar to the functional aspects discussed in the context of brightness adaptation.

However, the neuronal representation of brightness and motion differ in at least one fundamental aspect, leading us to suggest an additional novel functional role of motion adaptation. Unlike local brightness, local motion is represented by four subtypes of local motion-sensitive neurons each tuned to one of four cardinal directions (Borst, 2014; Borst et al., 2020). If motion adaptation were strongly dependent on the direction of motion, the sensitivity of the different subtypes of neurons would be affected in different ways depending on the direction of self-motion of the animal and the resulting optical flow. The direction of motion represented by these neurons would shift, even if the retinal motion vector remained the same. According to the results presented, local motion adaptation is induced by both preferred and null-direction motion of the movement detectors. In other experiments it has been shown that strong motion adaptation is also induced by motion orthogonal to these directions (Harris et al., 2000; Li, 2019; Niemeier, unpublished results). Therefore, these experiments together with those of the current study, all based on the activity of the spatially pooling HSE-cell, suggest that local motion adaptation is largely direction independent. Based on the modeling results of our present and previous studies (Li et al., 2017), this conclusion can be interpreted computationally by assuming that local motion adaptation takes place at the level of the half-detectors, which are direction sensitive to some extent. The direction independence of the adaptation is assumed to be accomplished by dividing the half-detector outputs by the temporally low-pass filtered and summated output signals of the PD and ND half-detectors of the ON and OFF pathways for both horizontal and vertical motion (see Figure 2C right panel). Accordingly, the adaptive state of the four half-detectors and, thus, the direction of the movement vector (though not its length) signaled by their joint activity would be invariant against the adaptive state of the motion vision pathway up to the level of local movement detection. The direct consequence would be a robust representation of the direction of local motion by the horizontal and vertical movement detectors irrespective of the overall direction of the optic flow generated by self-motion of the animal. This hypothesis needs to be further validated by experimental as well as simulation evidence.

We have refrained here from assigning the individual elements of the computational model to specific local interneurons in the visual motion pathway. Although the properties of important cellular elements involved in motion detection is increasingly being elucidated thanks to innovative genetic, imaging, and electrophysiological approaches (Mauss et al., 2017; Yang and Clandinin, 2018; Borst et al., 2020) mapping the individual computations of motion detection to anatomically and partly physiologically characterized local interneurons would currently be highly speculative. However, such a mapping is not necessary for our functional conclusions with regard to local or global movement adaptation.

The local motion-sensitive cells are then spatially pooled by the population of LTPCs, which have either a predominantly horizontal or vertical preferred direction (Hausen, 1984). Because of the strong direction dependent adaptation component operating at the level of LPTCs, the adaptive state of the population of these neurons is not adjusted in the same way by motion adaptation but depends on the way the animal has been moving. Hence, the self-motion vector represented by these neurons is not robust but depends on the way the animal moved before. The functional significance of this feature is not yet clear. It might be hypothesized that representing the direction of the vector of self-motion by the population of LPTCs is not of great functional relevance, as these cells are thought to mediate under closed-loop compensatory adjustments of the flight course after a disturbance. However, these cells have also been suggested to play a role in spatial vision, since they represent information about the spatial layout of the environment during the straight flight sections, i.e., during translatory motion. Then the responses of these cells do not only depend on the animal’s speed of locomotion, but also on the spatial profile of the environment. The responses increase when the animal passes nearby objects, because these lead to a larger retinal velocity than background structures in the environment. Note, that as a consequence of the saccadic flight and gaze strategy of flies and other insects translatory flight sections make up approximately 80% of flight time, whereas changes in flight directions are squeezed into rapid saccadic turns (reviews: Egelhaaf et al., 2012; Egelhaaf et al., 2014). Further analyses are required to clarify whether direction dependent motion adaptation of LTPCs plays a functional role in spatial vision.

## Data Availability Statement

The raw data supporting the conclusions of this article will be made available by the authors, without undue reservation.

## Author Contributions

JL, MN, RK, and ME conceptualized and designed the project and wrote the manuscript. JL performed the preliminary electrophysiological experiments, developed the model, and conducted the model simulations. MN collected the electrophysiological data shown in this study. JL and MN analyzed the data. All authors contributed to the article and approved the submitted version.

## Conflict of Interest

The authors declare that the research was conducted in the absence of any commercial or financial relationships that could be construed as a potential conflict of interest.

## Publisher’s Note

All claims expressed in this article are solely those of the authors and do not necessarily represent those of their affiliated organizations, or those of the publisher, the editors and the reviewers. Any product that may be evaluated in this article, or claim that may be made by its manufacturer, is not guaranteed or endorsed by the publisher.

## Footnotes

1
https://www.videolan.org
